# Crystallographic information data of natural occurring zaccariniite (RhNiAs) obtained by means of precession electron diffraction

**DOI:** 10.1016/j.dib.2019.104346

**Published:** 2019-08-02

**Authors:** Josep Roqué Rosell, Joaquim Portillo Serra, Thomas Hans Aiglsperger, Sergi Plana-Ruiz, Partha Pratim Das, Joan Mendoza Gonzalvez, Trifon Trifonov, Joaquin Antonio Proenza

**Affiliations:** a Department of Mineralogy, Petrology and Applied Geology, University of Barcelona, Marti i Franquès s/n, Barcelona, Catalunya, 08028, Spain; b Institut de Nanociència i Nanotecnologia, IN2UB Facultat de Química, Universitat de Barcelona Av. Diagonal 645, Barcelona, Catalunya, 08028, Spain; c Centres Científics i Tecnològics, Universitat de Barcelona, Lluís Solé i Sabaris, 1-3, Barcelona, Catalunya, 08028, Spain; d NanoMEGAS, Boulevard Edmond Machtens 79, Brussels, B-1080, Belgium; e Luleå University of Technology, Department of Civil Engineering and Natural Resources, Division of Geosciences and Environmental Engineering, SE, 97187, Luleå, Sweden; f Institut für Angewandte Geowissenschaften, Technische Universität Darmstadt, Schnittspahnstraße 9, 64287, Darmstadt, Germany; g Centre de Recerca en Ciència i Enginyeria Multiescala de Barcelona, Universitat Politècnica de Catalunya (UPC), Campus Diagonal Besòs, EEBE Eduard Maristany 10-14, Edifici I, planta S1, Catalunya, 08930, Sant Adrià de Besòs, Spain

**Keywords:** PED, RhNiAs, Zaccariniite, Crystallographic infromation file

## Abstract

The crystal structure of naturally occurring zaccariniite (RhNiAs) has been studied in Transmission Electron Microscopy (TEM) with variable angle Precession Electron Diffraction (PED) techniques. The analysis of the data has yielded tetragonal cell parameters of 3.86, 3.86, 6.77 Å and space group of P4/nmm for the basic structure, and its constituent atom positions for Ni, As and Rh were determined as well by ab-initio structure resolution method. The data is related to “Structural characterization and ab-initio resolution of natural occurring zaccariniite (RhNiAs) by means of Precession Electron Diffraction” (Roqué Rosell et al., 2019).

**Table:** Specifications Table

Subject	Geochemistry and Petrology
Specific subject area	Mineralogy Electron Crystallography
Type of data	Image Table Crystallographic information in CIF format
How data were acquired	Transmission Electron Microscopy (TEM) JEOL JEM 2100 LaB6 manufactured by JEOL Ltd. Precession Electron Diffraction (PED) system DigiSTAR™ manufactured by NanoMEGAS®
Data format	Raw Analyzed
Parameters for data collection	The acquired PED were obtained precessing the electron beam at 100Hz with the microscope column set in the TEM alfa 3 condenser illumination mode for 200kV. The TEM projector system operated in SAED mode with a virtual 50nm aperture to isolate the PED patterns corresponding to zaccariniite. These were recorded at 0.7° precession angle using an external high frame rate (50 frames per second) and low resolution (144 by 144 pixel at 8 bit) external optical camera set-up. Subsequently a tomographical 0.7° PED crystal tilting series, starting from an initial off-zone axis crystal tilt of −30° with respect to horizontal plane and acquiring and tilting the successive precession EDT patterns every +1°.
Description of data collection	Ab-initio structure resolution using PED.
Data source location	Department of Mineralogy, Petrology and Applied Geology, University of Barcelona, Marti i Franquès s/n, Barcelona, Catalunya, 08028, Spain.
Data accessibility	With the article
Related research article	J. Roqué Rosell, J. Portillo Serra, T.H. Aiglsperger, S. Plana Ruiz, P.P. Das, J. Mendoza Gonzalvez, T. Trifonov, J.A. Proenza, Structural characterization and ab-initio resolution of natural occurring zaccariniite (RhNiAs) by means of Precession Electron Diffraction, Microchem. J. 148 (2019) 130–140. https://doi.org/10.1016/j.microc.2019.04.071

**Table:** 

**Value of the data** • The data presented here is acquired using PED and presents a larger crystal cell than one obtained from the synthetic analogous zaccariniite in [2]. Therefore for the first time accurate crystal parameters of a natural occurring specimen of zaccariniite have been obtained. • The crystallographic data provided is expected to help in exploring and understanding Platinum-group minerals in earth and planetary sciences. Up to now these minerals were considered too small and heterogeneous to be able to be characterized using conventional techniques [1].

## 1 Data

**Fig. 1:**
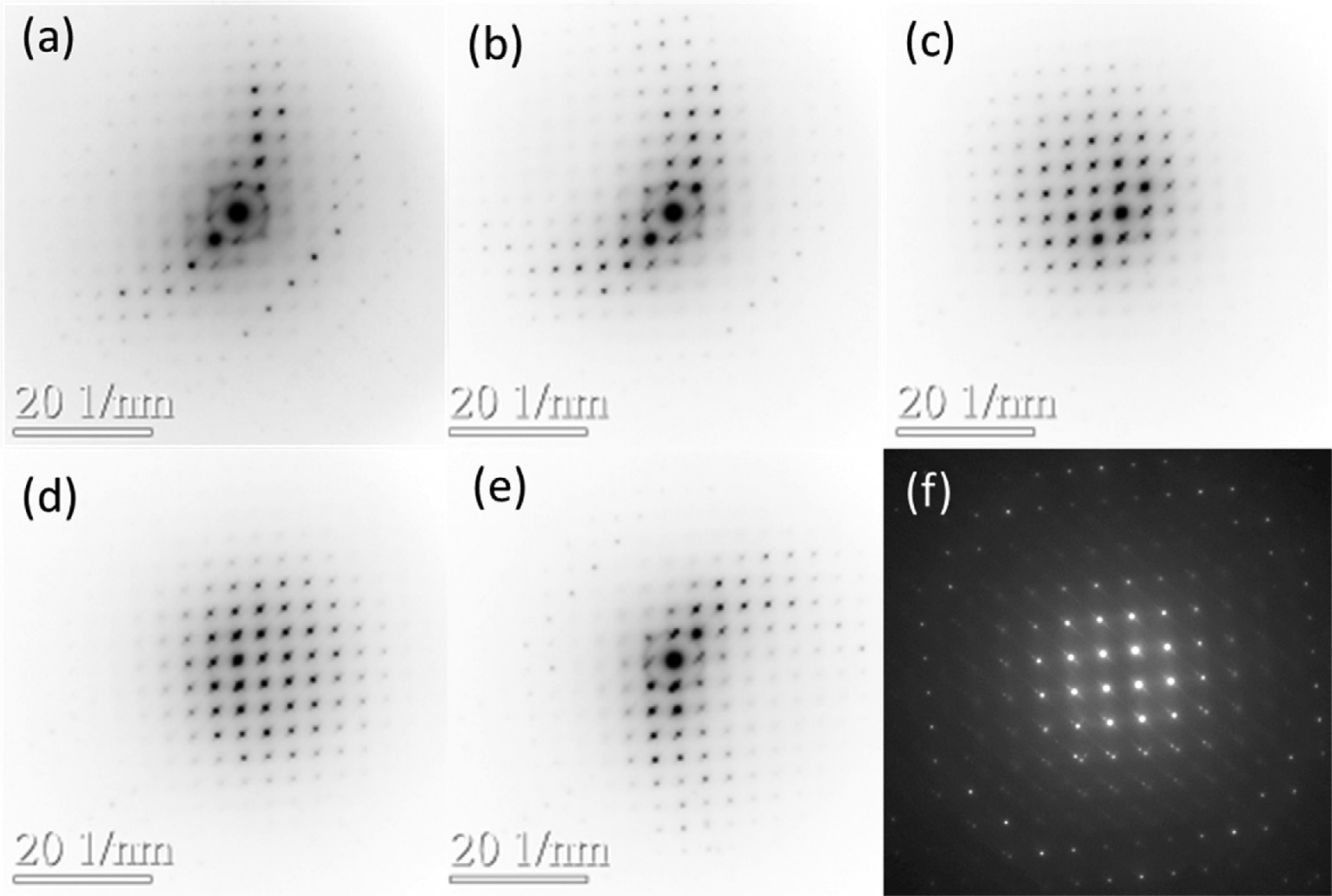
PED corresponding to (a,b,c,d, e) ZOLZ patterns obtained by means of Precession EDT (f) FOLZ pattern obtained by means of ZA high angle PED from zaccariniite [112]. The Precession EDT patterns have been used to extract the intensities to solve the zaccariniite structure ab initio using direct methods with SIR2014 [2].

The shared raw and processed data in the supplementary section correspond to the recordings of individual PED patterns using an external high frame rate optical camera and the corresponding extracted intensities (Fig. 1). The whole data was obtained on a natural occurring zaccariniite from a complex and heterogeneous mineral aggregate. The crystal structure of the natural zaccariniite is also presented from the ab-initio structure determination using the extracted PED intensities file on SIR2014 [1], [3]. The additional supporting information corresponding to the complete zaccariniite crystallographic structure is provided in the obtained.cif file format.

## 2 Experimental design, materials, and methods

A natural occurring zaccariniite electro transparent lamella was obtained using Focused Ion Beam Zeiss Neon40 and has been studied using a TEM JEOL JEM 2100 LaB6 at 200kV equipped with the precession system DigiSTAR manufactured by NanoMEGAS®. The intensities list used for structure determination were acquired from electron diffraction patterns obtained at 0.7° from Precession Electron Diffraction Tomography (Precession EDT) and 1.2°–2.2° from Zone Axis High Angle Precession Electron Diffraction (ZA high angle PED) (Fig. 1) [1]. The electron diffraction patterns processing to obtain the zaccariniite crystal structure is explained in [1].

The precession EDT intensities list offers a higher coverage of the 3D reciprocal space, compared to the obtained by means of ZA-PED, but the internal distribution of intensities in the former is not necessarily as quasi-kinematically as in the latter. The analysis of the data has yielded tetragonal cell parameters of 3.86, 3.86, 6.77 Å and space group of P4/nmm for the zaccariniite basic structure, and its constituent atom positions for Ni, As and Rh were determined as well by ab-initio structure resolution method using SIR2014 [1], [3]. The structure resolution using ZA PED intensity list provided a large thermal factor for the Ni atoms whereas the precession EDT intensity list provided balanced, low value thermal factors for all 3 atoms in the asymmetrical unit cell and with a remarkably low residual of 19.7% in trial 20 using 173 reflections. The raw data corresponding to the acquired PED patterns and the extracted intensities list are provided with the article and the resulting zaccariniite structure from trial 20 is presented in the attached.cif file.
